# Determinants of fungal infection and hospital readmission risk in interstitial pneumonia with autoimmune features: associations with vitamin D and pirfenidone

**DOI:** 10.3389/fimmu.2026.1825951

**Published:** 2026-08-03

**Authors:** Gaodi Yuan, Xiufang Xie, Min Tang, Xiaoli Zheng, Xiongyan Luo, Anji Xiong

**Affiliations:** 1 Department of Rheumatology and Immunology, The First People’s Hospital of Neijiang, Neijiang, Sichuan, China; 2 North Sichuan Medical College, Nanchong, Sichuan, China; 3 Department of Rheumatology and Immunology, Nanchong Central Hospital, The Affiliated Nanchong Central Hospital of North Sichuan Medical College, Nanchong Hospital of Beijing Anzhen Hospital Capital Medical University, Nanchong, Sichuan, China; 4 Department of Respiratory and Critical Care Medicine, The First People’s Hospital of Neijiang, Neijiang, Sichuan, China; 5 Department of Rheumatology and Immunology, the Affiliated Hospital of Southwest Medical University, Luzhou, Sichuan, China; 6 School of Basic Medicine, Southwest Medical University, Luzhou, Sichuan, China; 7 Department of Rheumatology and Immunology, West China Hospital, Sichuan University, Chengdu, Sichuan, China

**Keywords:** fungal infection, hospital readmission, interstitial pneumonia with autoimmune features, pirfenidone, risk factors, vitamin D

## Abstract

**Background:**

Fungal infections significantly compromise the prognosis of patients with interstitial pneumonia with autoimmune features (IPAF). However, the specific immune-related risk factors and their impact on clinical stability remain poorly defined. This study aimed to identify independent predictors for fungal infection and early readmission to optimize risk stratification.

**Methods:**

We conducted a retrospective analysis of 98 patients meeting the 2015 European Respiratory Society/American Thoracic Society (ERS/ATS) IPAF classification criteria. Fungal infections were confirmed through clinical manifestations, radiological findings, and metagenomic next-generation sequencing (mNGS). Logistic and Cox regression models were employed to identify factors independently associated with fungal infection and hospital readmission.

**Results:**

Fungal infection was identified in 40.8% of the cohort, with Candida albicans as the primary pathogen. Respiratory failure (odds ratio [OR]=3.76, 95% confidence interval [CI]: 1.24-11.38) and hypertension (OR = 2.94, 95% CI: 1.01-8.64) were independent associated with higher risks of fungal infection. Vitamin D (OR = 0.94, 95% CI: 0.89-0.99) and pirfenidone (OR = 0.17, 95% CI: 0.04-0.71) were independently associated with lower risks of fungal infection. Regarding prognosis, anti-Ro-52 (hazard ratio [HR]=2.23, 95% CI: 1.06-4.68) and anti-PL-12 (HR = 3.87, 95% CI: 1.11-13.44) antibody positivity independently predicted 3-month and 6-month hospital readmission, respectively.

**Conclusion:**

Fungal infections in IPAF involve a complex interplay between clinical comorbidities and immune status. In this single-center retrospective cohort, vitamin D and pirfenidone were independently associated with lower risks of fungal infection and hospital readmission after adjustment for confounders. These findings should be interpreted as associations rather than evidence of causality and require validation through large-scale, multicenter prospective studies.

## 1 Introduction

Interstitial pneumonia with autoimmune features (IPAF) is a special clinical phenotype of interstitial pneumonia, which falls between idiopathic interstitial lung disease (IIPs) and connective tissue disease-related interstitial lung disease (CTD-ILD)(1). It exhibits immune manifestations associated with connective tissue disease (CTD), but does not meet specific diagnostic criteria for CTD (1–4). This concept was proposed by the European Respiratory Society (ERS) and the American Thoracic Society (ATS) in 2015 to identify patients with subclinical autoimmune features (1). As research deepens, IPAF is gradually recognized as a unique phenotype, and some patients may develop clear CTD over time (5).

Growing evidence indicates the formation of a bidirectional pathogenic cycle between autoimmune activation and fungal colonization. On one hand, fungal antigens drive autoimmune dysregulation via the Th17/IL-23 axis, a critical pathway for maintaining immune homeostasis (6). Conversely, the use of immunosuppressive agents, such as corticosteroids, in patients with autoimmune diseases compromises mucosal antifungal defenses, increasing susceptibility to infection (7). Consequently, patients with IPAF experience recurrent autoimmune activation inherent to their disease while simultaneously requiring long-term immunosuppressive therapy. This subjects the immune system to persistent oscillation between hyperactivation and sustained suppression, ultimately manifesting as a progressive decline in antifungal capacity. As a result, the incidence of invasive fungal infections is significantly elevated, with Pneumocystis jirovecii pneumonia, aspergillosis, and candidiasis being particularly prominent (6, 8–10).

Although the risk of pulmonary fungal infection is significantly increased in patients with interstitial pneumonia with impaired immune function (11), current risk stratification tools primarily target CTD-ILD or transplant populations and fail to adequately consider the immune characteristics of IPAF patients. This highlights the urgent need for in-depth research into the risk factors for fungal infection in IPAF patients to provide more precise risk assessment for clinical decision-making (9, 12).

Vitamin D and pirfenidone may be clinically relevant to both infection susceptibility and hospital readmission in IPAF. Vitamin D participates in innate and adaptive immune regulation, helps maintain epithelial barrier integrity, and has been reported to influence antifungal responses through effects on fungal adhesion, biofilm formation, oxidative stress, and inflammatory balance (13, 14). Vitamin D may also be involved in pulmonary fibrotic remodeling through local activation in lung tissue and regulation of fibroblast activation and collagen deposition (15, 16). Pirfenidone is an antifibrotic agent that can inhibit transforming growth factor-beta (TGF-beta)-related profibrotic signaling, collagen deposition, and fibroblast activation (17–19). Because progressive fibrosis may contribute to impaired ventilation, hypoxic areas, altered mucociliary clearance, and local immune dysfunction, attenuation of fibrotic remodeling may partly improve the pulmonary microenvironment and reduce clinical instability (20). However, these mechanistic links remain exploratory in IPAF and require further prospective validation.

This study aimed to systematically investigate factors associated with fungal infections in IPAF patients, including immune status, clinical characteristics, and treatment strategies. Additionally, this study explored factors associated with hospital readmission in IPAF patients. By identifying these factors, this study sought to provide evidence for clinical risk stratification and to support optimized management strategies for IPAF patients.

## 2 Materials and methods

### 2.1 Study design and population

This retrospective observational study aimed to evaluate risk factors influencing fungal infection and readmission risks in patients with IPAF. We retrospectively screened medical records of 456 interstitial pneumonia patients hospitalized in the Department of Rheumatology and Immunology at Nanchong Hospital, Affiliated Anzhen Hospital of Capital Medical University, between 2019 and 2025. Ultimately, 98 patients meeting the diagnostic criteria for IPAF were included in the analysis.

### 2.2 Diagnostic criteria and exclusion criteria

All enrolled patients met the 2013 ATS/ERS diagnostic criteria for IIPs and the 2015 IPAF classification criteria (21, 22). Patient screening applied the following exclusion criteria: Connective tissue disease-associated interstitial pneumonia (n=122), Idiopathic interstitial pneumonia (n=72), Comorbid malignancies (n=6), Other interstitial pneumonias not meeting IPAF criteria (n=158).

### 2.3 Data collection and variable definitions

Baseline characteristics including demographic data, clinical comorbidities, laboratory results, and treatment regimens were retrospectively extracted from electronic medical records. Interstitial pneumonia diagnosis was primarily based on high-resolution computed tomography (HRCT) findings, which served to: Exclude other pulmonary pathologies and determine specific interstitial pneumonia patterns (e.g., non-specific interstitial pneumonia (NSIP), organizing pneumonia (OP), lymphocytic Interstitial Pneumonia (LIP), common interstitial pneumonia (UIP)).

#### 2.3.1 Fungal infection diagnosis

In this study, the diagnosis of fungal infection was primarily confirmed through the detection of fungal DNA or RNA in sputum samples using metagenomic next-generation sequencing (mNGS). To minimize interference from oral colonization, all patients followed a standardized sampling procedure and were instructed to rinse their mouths with nystatin solution before sputum collection. Sputum samples were then processed using mNGS technology, which allows high-throughput detection of microbial DNA/RNA and precise identification of various pathogens, including fungi. A positive result for fungal DNA or RNA was considered indicative of fungal colonization or infection. However, fungal infection was only diagnosed when positive mNGS results were supported by compatible clinical manifestations and radiological findings. This approach was used to improve the identification of clinically relevant fungal infections while reducing false-positive results related to colonization.

### 2.4 Follow up and data recording

All patients were followed for at least 6 months, during which fungal infection and hospital readmission due to worsening disease were recorded. In this study, hospital readmission was defined as readmission within 3 months or 6 months after discharge due to acute exacerbation or progression of IPAF-related interstitial lung disease symptoms. The assessment of hospital readmission was primarily based on clinical worsening and high-resolution computed tomography (HRCT) findings suggestive of acute exacerbation or progression of interstitial lung disease. No missing data were observed for the primary outcomes.

### 2.5 Statistical analysis

All statistical analyses were performed using SPSS software version 26.0 (IBM Corp., Armonk, NY, USA) and GraphPad Prism version 9.0 (GraphPad Software, San Diego, CA, USA). Continuous variables were tested for normality and presented as mean +/- standard deviation (SD) or median (interquartile range, IQR). Categorical variables were summarized as frequencies and percentages. Between-group comparisons were performed using the independent-samples t test or Mann-Whitney U test for continuous variables and the chi-square test or Fisher’s exact test for categorical variables. Logistic regression was used to evaluate factors associated with fungal infection, and Cox proportional hazards regression was used to analyze factors associated with hospital readmission. Univariable models were first fitted to describe crude associations between individual variables and outcomes. Covariates incorporated into the models were selected based on clinical risk factors and published literature evidence, rather than solely relying on P values from univariate analyses. Multivariable models were subsequently constructed to evaluate independent associations while accounting for potential confounding. Logistic regression results are reported as odds ratios (ORs) with 95% confidence intervals (CIs). Cox regression results are reported as hazard ratios (HRs) with 95% CIs. Multicollinearity was assessed using the variance inflation factor (VIF), with a VIF cutoff of 5. All variables had VIF values below 5, indicating that no significant multicollinearity was detected. A two-tailed P value <0.05 was considered statistically significant. The detailed VIF values for each variable in each regression model are provided in **Supplementary Tables 1-3**.

### 2.6 Ethics statement

The study involving human participants was approved by the Ethics Committee of Nanchong Central Hospital, The Affiliated Nanchong Central Hospital of North Sichuan Medical College, Nanchong Hospital of Beijing Anzhen Hospital Capital Medical University, Nanchong, Sichuan, China (approval No. 2025-149). The study was conducted in accordance with the Declaration of Helsinki, local legislation, and institutional requirements. Because this was a retrospective analysis of medical records, written informed consent was waived in accordance with national legislation and institutional requirements.

## 3 Results

### 3.1 Clinical characteristics of IPAF patients

This study included 98 patients with interstitial pneumonia with autoimmune features who underwent mNGS testing. Patients with positive fungal sequencing results and compatible clinical and radiological evidence were assigned to the fungal-positive group (n=40), while those without fungal infection were assigned to the fungal-negative group (n=58). The demographic characteristics, clinical manifestations, comorbidities, laboratory data, and treatment status of the two groups are presented in **Table 1**. The two groups had similar age and sex distributions, with a predominance of female patients. Regarding comorbidities, respiratory failure was more common in the fungal-positive group (55.00% vs 29.31%, P = 0.011), and hypertension was also more common in the fungal-positive group (37.50% vs 17.24%, P = 0.024). Compared with the fungal-negative group, patients in the fungal-positive group had lower median albumin levels (39.50 vs 40.90, P = 0.036) and higher ferritin levels (342.00 vs 194.50, P = 0.003). Both groups had relatively low vitamin D levels, and the fungal-positive group had significantly lower levels (18.48 vs 21.57, P = 0.015). There was no significant difference in the distribution of NSIP, UIP, OP, or LIP between groups. Treatment-related variables, including glucocorticoids, overall immunosuppressive therapy, cyclophosphamide, tacrolimus, mycophenolate mofetil, pirfenidone, and nintedanib, are stratified by group in **Table 1**.

**Table 1:** Characteristics of IPAF patients with and without fungal infection.

Variables	Fungal (-) n=58	Fungal (+) n=40	P value
Age, mean +_ SD, years	57.72 ± 12.73	60.50 ± 11.62	0.275
Male, n %	24 (41.38)	13 (32.50)	0.373
Clinical symptoms, n %			
Cough	46 (79.31)	35 (87.50)	0.293
Fever	8 (13.79)	7 (17.50)	0.616
Arthritis	5 (8.62)	2 (5.00)	0.776
Raynaud’s Disease	2 (3.45)	4 (10.00)	0.368
Morning Stiffness	2 (3.45)	3 (7.50)	0.668
Mechanic’s Hands	1 (1.72)		
Comorbidity, n %		–	–
Diabetes mellitus	16 (27.59)	14 (35.00)	0.434
Hypertension	10 (17.24)	15 (37.50)	0.024
Respiratory failure	17 (29.31)	22 (55.00)	0.011
pulmonary arterial hypertension	9 (15.52)	4 (10.00)	0.429
Osteoporosis	37 (63.79)	26 (65.00)	0.902
Emphysema	15 (25.86)	10 (25.00)	0.923
Hyperlipidemia	23 (39.66)	13 (32.50)	0.470
Laboratory indicators			
Lymphocyte count	1.57 (1.31, 2.03)	1.50 (1.12, 1.82)	0.392
LDH, median (IQR), IU/L	222.80 (189.35, 283.20)	254.10 (215.50, 296.15)	0.166
CRP, median (IQR), mg/dl	2.10 (0.70, 7.32)	3.61 (1.06, 8.99)	0.315
FET, median (IQR), ng/ml	194.50 (101.00, 341.25)	342.00 (195.00, 583.25)	0.003
HDL, median (IQR), ng/ml	1.33 (1.19, 1.59)	1.44 (1.05, 1.68)	0.876
Glucose, median (IQR), ng/ml	5.46 (4.73, 6.65)	5.15 (4.26, 5.86)	0.151
Sodium, median (IQR), ng/ml	141.90 (140.22, 143.07)	140.90 (138.55, 143.00)	0.180
Vitamin D, median (IQR), ng/ml	21.57 (17.39, 32.39)	18.48 (14.93, 23.52)	0.015
Albumin median (IQR), ng/ml	40.90 (37.38, 43.48)	39.50 (35.98, 41.35)	0.036
Radiological findings, n %			
NSIP	36 (62.07)	25 (62.50)	0.965
OP	11 (18.97)	8 (20.00)	0.899
LIP	4 (6.90)	–	–
UIP	7 (12.07)	7 (17.50)	0.450
Treatment, n %			
GCs	24 (41.38)	23 (57.50)	0.116
IS	14 (24.14)	10 (25.00)	0.922
CYC	6 (10.34)	4 (10.00)	1.000
TAC	1 (1.72)	3 (7.50)	0.368
MMF	3 (5.17)	5 (12.50)	0.354
Pirfenidone	15 (25.86)	5 (12.50)	0.107
Nintedanib	9 (15.52)	6 (15.00)	0.944
Autoantibody, n %			
Anti-Dsdna, positive	3 (5.17)	7 (17.50)	0.101
ANA 1:100, positive	14 (24.14)	10 (25.00)	0.922
ANA 1:320, positive	17 (29.31)	15 (37.50)	0.395
Anti-RO-52, positive	17 (29.31)	13 (32.50)	0.736
Anti-PL-12, positive	3 (5.17)	1 (2.50)	0.890
Anti-PL-7, positive	6 (10.34)	2 (5.00)	0.566
RF, positive	4 (6.90)	2 (5.00)	1.000
Anti-MDA5, positive	1 (1.72)	2 (5.00)	0.742
Anti-JO-1, positive	7 (12.07)	4 (10.00)	1.000
Clinical outcomes, n %			
Three-months readmission	21(36.21)	13(32.50)	0.705
Six-months readmission	22(37.93)	18(45.00)	0.484

SD, standard deviation; IQR, interquartile range; CRP, C-reactive protein; FET, ferritin; HDL, high-density lipoprotein; NSIP, nonspecific interstitial pneumonia; OP, organizing pneumonia; LIP, lymphoid interstitial pneumonia; UIP, usual interstitial pneumonia; GCs, glucocorticoids; IS, immunosuppressive therapy; CYC, cyclophosphamide; TAC, tacrolimus; MMF, mycophenolate mofetil. A dash indicates that no event was observed in that subgroup; related P values were not calculated when appropriate. Bold values indicate statistical significance for 95% confidence intervals and p-values (p < 0.05).

### 3.2 Risk factors for fungal infection of IPAF patients

To explore factors associated with fungal infection in IPAF patients, we first conducted univariable logistic regression analyses to describe the crude associations between each clinical variable and the occurrence of fungal infection. In the univariable analysis, respiratory failure (OR = 3.26, 95% CI: 1.40-7.59) and hypertension (OR = 3.55, 95% CI: 1.41-8.95) were significantly associated with higher odds of fungal infection. Higher albumin levels (OR = 0.90, 95% CI: 0.82-0.99) and vitamin D levels (OR = 0.95, 95% CI: 0.90-0.99) were associated with lower odds of fungal infection. Pirfenidone showed a trend toward lower odds of fungal infection in the univariable model (OR = 0.41, 95% CI: 0.14-1.24), while anti-dsDNA antibodies demonstrated borderline significance (OR = 3.89, 95% CI: 0.94-16.08).

A multivariable logistic regression model was then constructed to estimate independent associations while adjusting for clinically relevant confounders. After adjustment, respiratory failure (OR = 3.76, 95% CI: 1.24-11.38) and hypertension (OR = 2.94, 95% CI: 1.01-8.64) remained significant independent risk factors for fungal infection. Vitamin D (OR = 0.94, 95% CI: 0.89-0.99) and pirfenidone (OR = 0.17, 95% CI: 0.04-0.71) were independently associated with lower odds of fungal infection. Albumin and anti-dsDNA antibodies remained borderline significant in the adjusted model. Detailed results are presented in **Table 2**.

**Table 2:** Risk factors for fungal infection occurrence in IPAF patients.

Variables	Univariable		Multivariable	
	OR (95% CI)	P value	OR (95% CI)	P value
Age	1.02 (0.99 ~ 1.05)	0.974	*	*
Male	0.68 (0.29 ~ 1.58)	0.184	*	*
Fever	1.33 (0.44 ~ 4.00)	0.617	*	*
Respiratory Failure	3.26 (1.40 ~ 7.59)	0.006	**3.76 (1.24 ~ 11.38)**	**0.019**
Hypertension	3.55 (1.41 ~ 8.95)	0.007	**2.94 (1.01 ~ 8.64)**	**0.049**
Lymphocyte count	0.79 (0.43 ~ 1.45)	0.450	*	*
LDH	1.00 (1.00 ~ 1.01)	0.658	*	*
CRP	1.00 (0.98 ~ 1.01)	0.747	*	*
FET	1.00 (1.00 ~ 1.00)	0.182	*	*
Albumin	0.90 (0.82 ~ 0.99)	0.026	0.92 (0.83 ~ 1.03)	0.144
Vitamin D	0.95 (0.90 ~ 0.99)	0.019	**0.94 (0.89 ~ 0.99)**	**0.029**
Glucose	0.92 (0.73 ~ 1.15)	0.446	*	*
Sodium	0.89 (0.78 ~ 1.02)	0.086	*	*
GCs	1.92 (0.85 ~ 4.33)	0.118	*	*
Pirfenidone	0.41 (0.14 ~ 1.24)	0.114	**0.17 (0.04 ~ 0.71)**	**0.014**
IS	1.05 (0.41 ~ 2.67)	0.922	*	*
Anti-Dsdna	3.89 (0.94 ~ 16.08)	0.061	3.90 (0.78 ~ 19.58)	0.098
Anti-Ro-52	1.16 (0.49 ~ 2.77)	0.736	*	*
Anti-PL-12	0.47 (0.05 ~ 4.69)	0.520	*	*

*Not included in the multivariable model.

CI, confidence interval; CRP, C-reactive protein; FET, ferritin; LDH, lactate dehydrogenase; HDL, high-density lipoprotein; GCs, glucocorticoids; TAC, tacrolimus; IS, immunosuppressive therapy; OR, odds ratio. Bold values indicate statistical significance for 95% confidence intervals and p-values (p < 0.05).

### 3.3 Risk factors for readmission of IPAF patients

**Figure 1:**
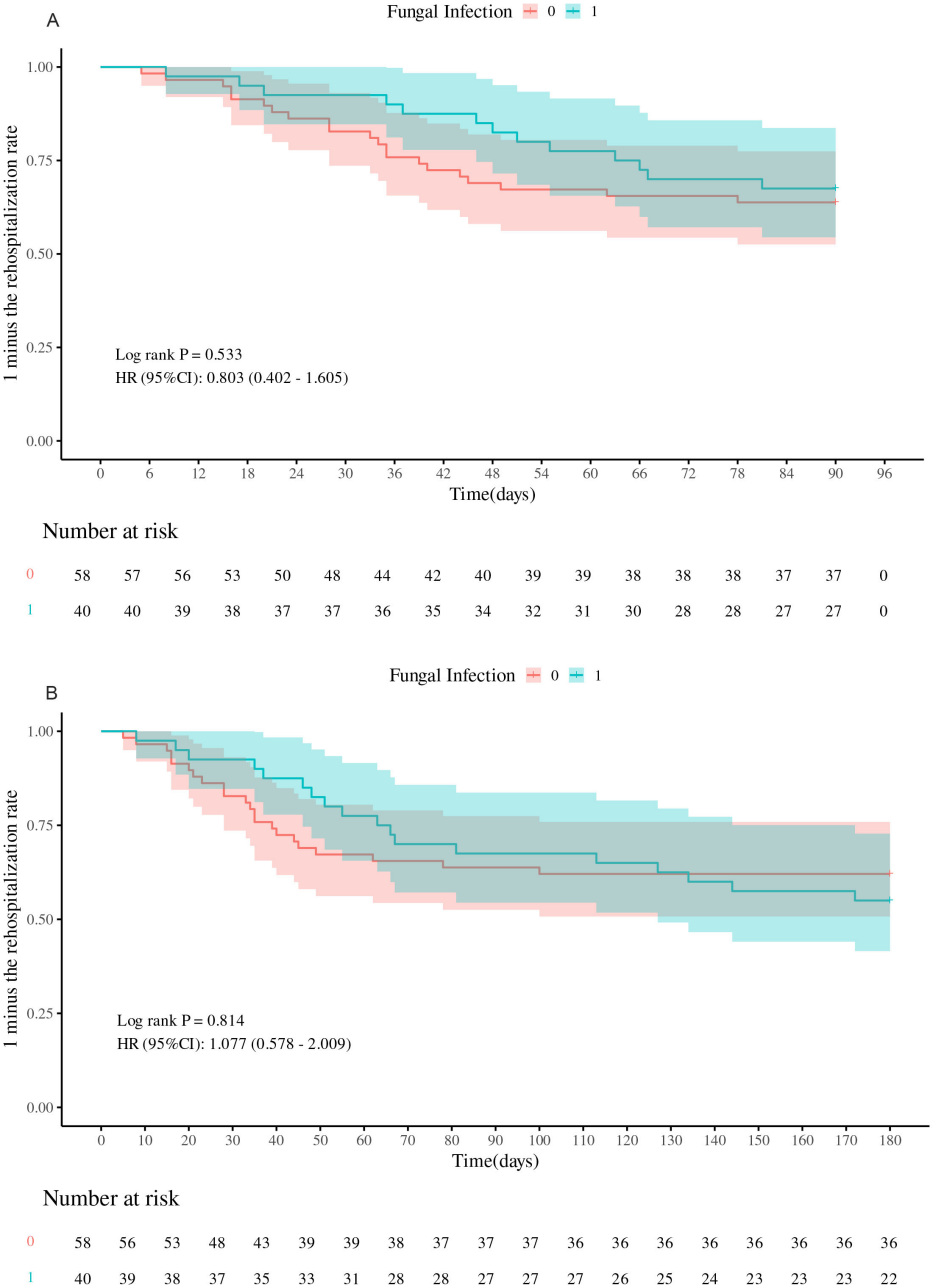
Kaplan-Meier curves comparing hospital readmission rates in IPAF patients with and without fungal infection over 90 days **(A)** and 180 days **(B)**. No statistically significant between-group differences were observed (log-rank P = 0.533 for 90 days; log-rank P = 0.814 for 180 days).

Within the first three months of follow-up, the overall hospital readmission rate among IPAF patients was 34.69%. As shown in **Figure 1A**, no statistically significant difference in cumulative 3-month hospital readmission was observed between patients with and without fungal infection (log-rank P = 0.533).

To identify factors associated with readmission, Cox proportional hazards regression was performed (**Table 3**). In the univariable analysis, anti-Ro-52 antibodies (HR = 2.07, 95% CI: 1.05–4.08), anti-PL-12 antibodies (HR = 4.16, 95% CI: 1.26–13.74), and elevated CRP levels (HR = 1.01, 95% CI: 1.01–1.02) were associated with an increased risk of readmission. Higher high-density lipoprotein (HDL) levels (HR = 0.34, 95% CI: 0.13–0.89) and vitamin D levels (HR = 0.95, 95% CI: 0.91–0.99) were associated with a decreased risk.

**Table 3:** Risk factors for hospital readmission within three months in IPAF patients.

Variables	Univariable		Multivariable	
	HR (95% CI)	P value	HR (95% CI)	P value
Age	1.02 (0.99 ~ 1.05)	0.974	*	*
Male	0.88 (0.44 ~ 1.78)	0.725	*	*
Fever	0.75 (0.26 ~ 2.12)	0.581	*	*
Respiratory Failure	0.93 (0.47 ~ 1.86)	0.840	*	*
Hypertension	0.84 (0.38 ~ 1.86)	0.669	*	*
Lymphocyte count	0.84 (0.51 ~ 1.40)	0.507	*	*
FET	1.00 (1.00 ~ 1.00)	0.351	*	*
CRP	1.01 (1.01 ~ 1.02)	0.038	1.01 (1.00 ~ 1.02)	0.091
HDL	0.34 (0.13 ~ 0.89)	0.028	**0.29 (0.10 ~ 0.80)**	**0.017**
Vitamin D	0.95 (0.91 ~ 0.99)	0.013	**0.96 (0.92 ~ 0.99)**	**0.031**
Glucose	0.92 (0.73 ~ 1.15)	0.446	1.11 (0.93 ~ 1.33)	0.242
GCs	1.13 (0.58 ~ 2.22)	0.715	*	*
Pirfenidone	0.32 (0.10 ~ 1.05)	0.060	**0.29 (0.09 ~ 0.96)**	**0.042**
TAC	2.89 (0.88 ~ 9.50)	0.081	*	*
Anti-DsDNA	1.44 (0.56 ~ 3.73)	0.449	*	*
Anti-Ro-52	2.07 (1.05 ~ 4.08)	0.035	**2.23 (1.06 ~ 4.68)**	**0.035**
Anti-PL-12	4.16 (1.26 ~ 13.74)	0.019	4.16 (0.98 ~ 17.62)	0.053
Anti-ANA	1.18 (0.58 ~ 2.39)	0.641	*	*

*Not included in the multivariable model.

CI, confidence interval; FET, ferritin; LDH, lactate dehydrogenase; HDL, high-density lipoprotein; GCs, glucocorticoids; TAC, tacrolimus; HR, hazard ratio. Bold values indicate statistical significance for 95% confidence intervals and p-values (p < 0.05).

After adjusting for clinically relevant confounders in the multivariable model, anti-Ro-52 antibodies remained an independent risk factor for readmission (HR = 2.23, 95% CI: 1.06–4.68). Conversely, pirfenidone use (HR = 0.29, 95% CI: 0.09–0.96), higher HDL levels (HR = 0.29, 95% CI: 0.10–0.80), and higher vitamin D levels (HR = 0.96, 95% CI: 0.92–0.99) were independently associated with a lower risk of readmission (**Table 3**).

When the observation period was extended to six months, the cumulative hospital readmission rate among IPAF patients increased to 40.82%. As shown in **Figure 1B**, no statistically significant difference in cumulative 6-month hospital readmission was observed between patients with and without fungal infection (log-rank P = 0.814).

Cox proportional hazards regression was then performed to identify factors associated with six-month readmission (**Table 4**). In the univariable analysis, anti-PL-12 antibodies (HR = 3.64, 95% CI: 1.11–11.90) were associated with a higher risk of readmission. In contrast, higher HDL levels (HR = 0.41, 95% CI: 0.17–0.98) and higher vitamin D levels (HR = 0.94, 95% CI: 0.90–0.98) were associated with a reduced risk.

**Table 4:** Risk factors for hospital readmission within six months in IPAF patients.

Variables	Univariable		Multivariable	
	HR (95% CI)	P value	HR (95% CI)	P value
Age	0.99 (0.97 ~ 1.02)	0.586	*	*
Male	0.77 (0.40 ~ 1.49)	0.436	*	*
Fever	0.78 (0.31 ~ 1.99)	0.605	*	*
Respiratory Failure	1.00 (0.53 ~ 1.88)	0.996	*	*
Hypertension	0.91 (0.45 ~ 1.86)	0.799	*	*
Lymphocyte count	0.86 (0.54 ~ 1.36)	0.521	*	*
FET	1.00 (1.00 ~ 1.00)	0.308	*	*
HDL	0.41 (0.17 ~ 0.98)	0.044	**0.34 (0.14 ~ 0.80)**	**0.014**
Vitamin D	0.94 (0.90 ~ 0.98)	0.002	**0.94 (0.91 ~ 0.98)**	**0.003**
GCs	1.26 (0.68 ~ 2.35)	0.464	*	*
Pirfenidone	0.46 (0.18 ~ 1.18)	0.106	0.42 (0.17 ~ 1.09)	0.075
TAC	2.62 (0.80 ~ 8.57)	0.110	*	*
Anti-DsDNA	1.25 (0.49 ~ 3.18)	0.646	*	*
Anti-Ro-52	1.81 (0.96 ~ 3.41)	0.067	1.68 (0.88 ~ 3.23)	0.119
Anti-PL-12	3.64 (1.11 ~ 11.90)	0.033	**3.87 (1.11 ~ 13.44)**	**0.033**
Anti-ANA	1.32 (0.69 ~ 2.50)	0.400	*	*

*Not included in the multivariable model.

CI, confidence interval; FET, ferritin; HDL, high-density lipoprotein; GCs, glucocorticoids; TAC, tacrolimus; HR, hazard ratio. Bold values indicate statistical significance for 95% confidence intervals and p-values (p < 0.05).

After adjusting for clinically relevant confounders, anti-PL-12 antibodies remained an independent risk factor for six-month readmission (HR = 3.87, 95% CI: 1.11-13.44). Vitamin D (HR = 0.94, 95% CI: 0.91-0.98) and HDL (HR = 0.34, 95% CI: 0.14-0.80) continued to demonstrate independent associations with lower risk of six-month readmission.

### 3.4 Fungal infection rate and co-infection status in IPAF patients

**Figure 2:**
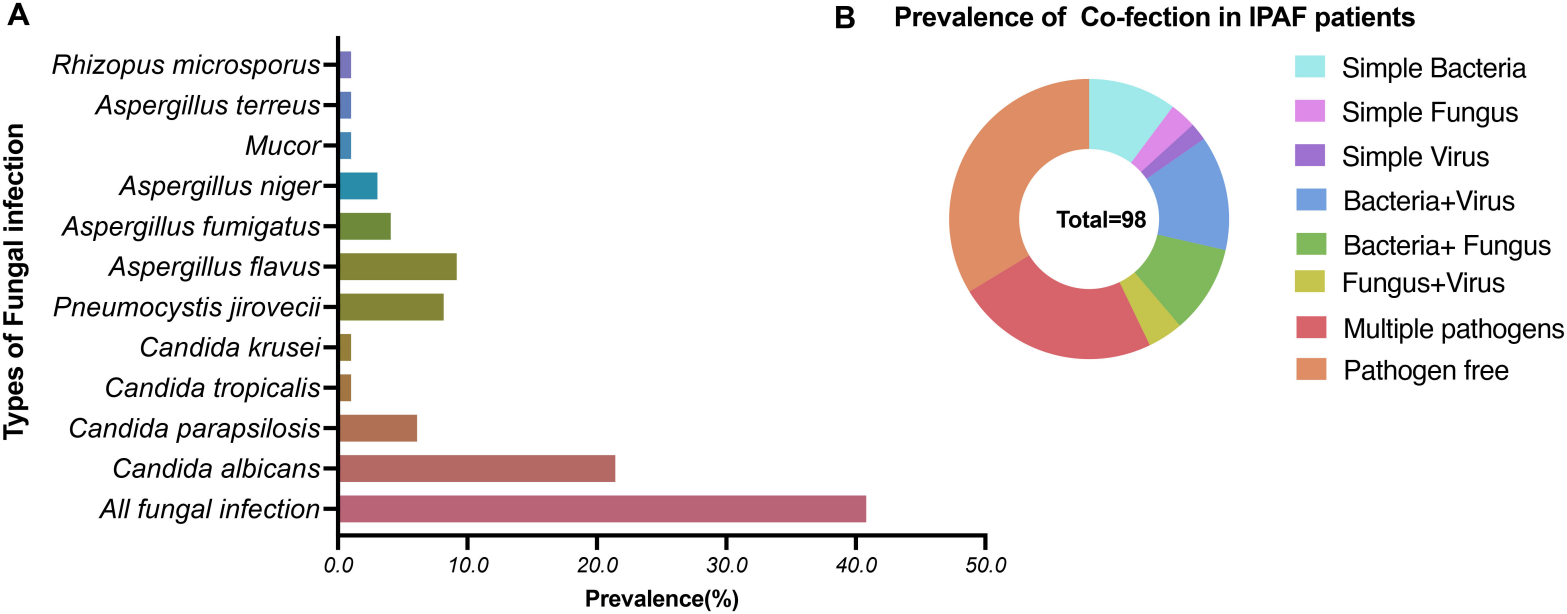
**(A)** Distribution of fungal infection types among IPAF patients, presented as absolute case numbers and percentages of the total cohort (N = 98). Candida albicans was the most common fungal pathogen. **(B)** Composition of co-infection types in IPAF patients (N = 98). Co-infections include combinations of bacteria, fungi, and viruses.

In this study, 40 patients were diagnosed with fungal infections, with an infection rate of 40.8%. Candida albicans was the most common fungal pathogen (21/98, 21.43%), followed by Aspergillus flavus (9/98, 9.18%), Pneumocystis jirovecii (8/98, 8.16%), Candida parapsilosis (6/98, 6.12%), Aspergillus fumigatus (4/98, 4.08%), and Aspergillus niger (3/98, 3.06%). Candida intermedia, Candida krusei, Rhizopus oryzae, other Aspergillus species, and Rhizopus microsporus were each detected in 1 patient (1/98, 1.02%) (**Figure 2A**).

An analysis of co-infections in IPAF patients revealed that co-infections are prevalent among IPAF patients, with a total of 51.02% of patients infected with two or more pathogens. Among the types of co-infections, the most common was a triple infection involving fungi, bacteria, and viruses, with an infection rate of 23.47%, followed by bacterial and viral co-infections (13.27%) and bacterial and fungal co-infections (10.2%) (**Figure 2B**).

## 4 Discussion

This study investigated factors associated with fungal infections in patients with IPAF and their association with hospital readmission risk. We found that the incidence of fungal infections was 40.8% among 98 IPAF patients, with Candida albicans being the most common pathogen. Co-infections involving fungi, bacteria, and viruses were prevalent among these patients, further complicating treatment. Respiratory failure and hypertension were independently associated with higher odds of fungal infection, while vitamin D levels and pirfenidone use were independently associated with lower odds of fungal infection. Although there was no significant difference in hospital readmission rates between the fungal-positive and fungal-negative groups at three and six months, anti-Ro-52 antibody positivity was independently associated with higher 3-month hospital readmission risk, and anti-PL-12 antibody positivity was independently associated with higher 6-month hospital readmission risk.

We found that respiratory failure and hypertension, as comorbidities in IPAF patients, significantly increased the risk of fungal infection. Therefore, clinically, for IPAF patients with respiratory failure, in addition to providing respiratory support, there should be enhanced monitoring and early intervention for fungal infections. Especially in patients requiring mechanical ventilation support, mechanical ventilation may further increase the risk of fungal infection by suppressing immune function and weakening pulmonary defense mechanisms (23). Hypertension, as a common chronic disease, although its direct relationship with fungal infections is not yet fully understood, has been shown in studies to potentially lead to immune cell dysfunction through hypertension-driven chronic inflammation, further exacerbating immune dysregulation (24). Therefore, in the context of immunosuppressive therapy, chronic hypertension may exacerbate immune dysregulation, thereby increasing the risk of infection. For IPAF patients, managing hypertension not only helps reduce cardiovascular risk but may also indirectly lower the incidence of infection by alleviating chronic inflammatory responses. In addition to IPAF complications, vitamin D levels play a significant role in preventing fungal infections and readmissions.

We found that relatively higher vitamin D levels were independently associated with lower risks of fungal infection and hospital readmission within three and six months in IPAF patients. The antifungal effects of vitamin D may be related to its lipophilic structure and immunomodulatory properties, which may help disrupt fungal cell membranes, reduce fungal adhesion and hydrophobicity, inhibit biofilm formation, affect cell wall integrity, and increase reactive oxygen species production (13, 14). Previous studies have reported associations between vitamin D deficiency and fungal infections, including Candida and pulmonary Aspergillus infections, which is broadly consistent with our findings (25–27). Regarding hospital readmission, vitamin D may also be relevant to pulmonary fibrosis biology. Vitamin D can be locally activated in lung tissue and may slow fibrotic progression by inhibiting pulmonary fibroblast activation and collagen deposition (15, 16, 28). Therefore, vitamin D status may have value for risk stratification in IPAF, although whether vitamin D supplementation can improve clinical outcomes requires prospective interventional studies.

In the present cohort, pirfenidone use was independently associated with lower odds of fungal infection and a lower risk of 3-month hospital readmission after adjustment for confounders. Pirfenidone may inhibit profibrotic mediators such as TGF-beta and tumor necrosis factor-alpha (TNF-alpha), reduce collagen deposition and fibroblast proliferation, and slow pulmonary fibrotic remodeling (17–19). Fibrotic lung tissue may be characterized by impaired ventilation, regional hypoxia, altered mucociliary clearance, and local immune dysregulation, which could facilitate fungal colonization or infection (20). By attenuating TGF-beta-related fibrotic remodeling and potentially improving the local pulmonary microenvironment, pirfenidone might be associated with reduced susceptibility to fungal infection. Nevertheless, direct evidence supporting an antifungal effect of pirfenidone in IPAF is limited; therefore, this interpretation should be considered hypothesis-generating and requires confirmation in future mechanistic and prospective studies.

In terms of IPAF biomarkers, we found that anti-Ro-52 antibody positivity was independently associated with increased risk of hospital readmission within three months, while anti-PL-12 antibody positivity was independently associated with increased risk of hospital readmission within six months. These antibodies may help clinicians identify patients at higher risk of short-term disease instability. In addition to these antibodies, higher HDL levels were independently associated with lower risks of hospital readmission within three and six months. However, because direct clinical evidence regarding HDL-targeted interventions in IPAF is lacking, the clinical value of HDL should be interpreted cautiously and further evaluated in future studies.

Although this study provides clinical insights into the management of IPAF, several limitations must be acknowledged. First, the retrospective, single-center design may introduce selection bias, and the sample size of 98 patients remains relatively small. This may limit the statistical power for subgroup analyses of rare fungal subtypes and pathogens, and the conclusions cannot be directly generalized to multicenter populations. Second, while mNGS offers high-resolution pathogen detection, distinguishing fungal colonization from active infection remains challenging. To mitigate this, all patients underwent standardized nystatin oral rinsing before sputum collection, and fungal infection was diagnosed only when positive mNGS results were supported by compatible clinical symptoms and progressive radiological findings. Third, the cumulative dosage and treatment duration of glucocorticoids and immunosuppressants were not recorded, which may lead to unadjusted confounding bias. Other vitamin intake and complete data on additional medications that may influence inflammation or immune status were also not systematically collected. Fourth, only total serum 25-hydroxyvitamin D [25(OH)D] was measured, without differentiation of active vitamin D metabolites; therefore, the optimal supplementation threshold cannot be determined. Finally, as a retrospective analysis, this study can establish associations but cannot confirm causal relationships between vitamin D, pirfenidone, and clinical outcomes. Prospective multicenter studies and interventional trials are still needed to validate these findings.

## 5 Conclusion

This single-center retrospective cohort study showed a high prevalence of fungal infections (40.8%) in IPAF patients and highlighted the potential value of mNGS for pathogen identification. Respiratory failure and hypertension were independently associated with increased risk of fungal infection. Vitamin D and pirfenidone were independently associated with lower risks of fungal infection and hospital readmission after adjustment for confounders, but these findings should not be interpreted as evidence of causality. Anti-Ro-52 and anti-PL-12 antibodies may serve as predictive biomarkers for short-term and longer-term hospital readmission risk, respectively. This study only identifies predictive biomarkers for risk stratification; prospective interventional trials are still required to validate whether vitamin D supplementation and pirfenidone treatment can improve long-term clinical outcomes of IPAF patients.

## Data Availability

The original contributions presented in the study are included in the article/**Supplementary Material**. Further inquiries can be directed to the corresponding authors.
