# Supplementary material for: Determinants of fungal infection and hospital readmission risk in interstitial pneumonia with autoimmune features: associations with vitamin D and pirfenidone
Source: Front Immunol. 2026 Aug 3;17:1825951. doi: 10.3389/fimmu.2026.1825951 (PMC13478266; doi:10.3389/fimmu.2026.1825951)
Supplement: Supplementary file 1 [file Table1.docx]

Table S1. VIF for Variables in the Fungal Infection Model

| **Variable** | **VIF** |
| --- | --- |
| Respiratory failure | 1.324 |
| Hypertension | 1.042 |
| Anti-Dsdna | 1.042 |
| Pirfenidone | 1.113 |
| Albumin | 1.172 |
| Vitamin D | 1.036 |

VIF: Variance Inflation Factors

Table S2. VIF for Variables in the Three-Month Readmission Model

| **Variable** | **VIF** |
| --- | --- |
| Anti-Ro52 | 1.025 |
| Anti-PL12 | 1.084 |
| CRP | 1.156 |
| Glucose | 1.145 |
| HDL | 1.157 |
| Vitamin D | 1.052 |

VIF: Variance Inflation Factors, **CRP: C-reactive protein, HDL: High-density lipoprotein,**

Table S3. VIF for Variables in the Six-Month Readmission Model

| **Variable** | **VIF** |
| --- | --- |
| Anti-PL12 | 1.050 |
| HDL | 1.019 |
| Vitamin D | 1.046 |
| Pirfenidone | 1.015 |
| Anti-RO52 | 1.046 |

VIF: Variance Inflation Factors, **HDL: High-density lipoprotein,**
